# Strategies of Increased Protein Intake in ELBW Infants Fed by Human Milk Lead to Long Term Benefits

**DOI:** 10.3389/fpubh.2018.00272

**Published:** 2018-09-27

**Authors:** Elisa Mariani, Augusto Biasini, Lucia Marvulli, Silvia Martini, Arianna Aceti, Giacomo Faldella, Luigi Corvaglia, Alessandra Sansavini, Silvia Savini, Francesca Agostini, Marcello Stella, Erica Neri

**Affiliations:** ^1^Pediatric and Neonatal Intensive Care Unit, M. Bufalini Hospital, Cesena, Italy; ^2^Donor Human Milk Bank Italian Association (AIBLUD), Milan, Italy; ^3^Neonatology and Neonatal Intensive Care Unit—S. Orsola-Malpighi Hospital, Bologna, Italy; ^4^Department of Psychology, University of Bologna, Bologna, Italy

**Keywords:** nutrition ELBW, protein intake, long term neurologic advantages, full feeding achievement, speed of growth

## Abstract

**Objective:** The aim of this observational study was to evaluate the effects of two different protein intake regimes on feeding tolerance, in-hospital growth, anthropometric data and psychomotor outcome up to 24 months corrected age (CA) in extremely low birth-weight (ELBW; birth weight <1000 g) infants.

**Methods:** During the period 2008–2013, 52 ELBW infants admitted at birth to two Neonatal Intensive Care Units of Emilia Romagna (Italy) were fed according to different protocols of protein fortification of human milk: an estimated protein intakes at maximum fortification levels of 3.5 gr/kg/day in the Standard Nutrition Population-SNP group (*n* = 26) and 4.8 g/kg/day in the Aggressive Nutrition Population-ANP group (*n* = 26). During hospitalization, infants' growth, biochemical indices of nutritional status, enteral intake, feeding tolerance, clinical history and morbidity were evaluated. After discharge, anthropometric data and psychomotor outcome, evaluated by Revised Griffiths Mental Development Scales (GMDS-R) 0–2 years, were assessed up to 24 months CA.

**Results:** During hospitalization, the ANP group showed significantly higher weight (18.87 vs. 15.20 g/kg/day) and head circumference (0.70 vs. 0.52 cm/week) growth rates compared to SNP, less days of parenteral nutrition (7.36 ± 2.7 vs. 37.75 ± 29.6) and of hospitalization (60.0 ± 13.3 vs. 78.08 ± 21.32). After discharge, ANP infants had a greater head circumference compared to SNP (45.64 ± 0.29; 46.80 ± 0.31). Furthermore, the General Quotient of GMDS-R mean scores in the SNP group significantly decreased from 12 to 24 months CA, while no difference was seen in the ANP group.

**Conclusions:** Increased protein intake may provide short and long term benefits in terms of growth and neurodevelopment in human milk-fed ELBW infants.

## Introduction

The main goals of preterm infants' nutrition are the achievement of postnatal growth rates similar to those of normal fetuses of the same gestational age, a mimic fetal body composition and neurodevelopmental outcomes comparable to term-born infants (1).

In-hospital weight, length, and head circumference (HC) growth rates are positively correlated with neurodevelopment and, possibly, with an improved brain growth and neurological maturation in the preterm population (2, 3). Conversely, extra-uterine growth restriction (EUGR), defined as weight, length, or HC ≤ 10th percentile of intra-uterine growth expectation for correspondent postmenstrual age at hospital discharge (4), is a negative prognostic factor for long-term neurodevelopment (2). Adequate nutrition during hospitalization is fundamental in order to prevent EUGR and to optimize long-term growth and neurodevelopment in the preterm population. However, due to their gastro-intestinal immaturity, very preterm infants often experience poor feeding tolerance during their stay in Neonatal Intensive Care Unit (NICU), and this contributes to hinder the achievement of optimal nutritional intakes over the first weeks of life (5). As a consequence, significant energy and nutrients deficits are frequently established during NICU stay, and inadequate protein and energy intakes may account for up to 45% of postnatal weight restriction in very-low-birth-weight preterm infants at hospital discharge (6, 7).

The beneficial effects of human milk feeding have been currently acknowledged to overcome the delayed weight gain associated to the lower protein and energy contents of human milk compared to formula (8, 9). However, it has been previously shown that actual protein intakes after standard protein fortification of human milk are substantially lower than those recommended over the first weeks of life (10, 11). Furthermore, data on the possible influence of protein intakes on neonatal growth and neurodevelopment in extremely low birth weight (ELBW) infants are still limited (12).

This study aimed to evaluate the effect of two nutritional approaches, providing different protein regimens, on in-hospital and post-discharge growth and psychomotor outcomes in ELBW preterm infants followed up to 24 months of corrected age (CA).

## Materials and methods

During the period 2008–2013, all preterm infants admitted to two level III NICUs of Emilia Romagna region (Italy), Sant'Orsola Malpighi Hospital NICU (Bologna) and Bufalini Hospital NICU (Cesena), were included in the present study if the following inclusion criteria were fulfilled: birth-weight (BW) <1000 g, gestational age (GA) <32 weeks, exclusive human milk feeding (own mother's milk [OMM] or donor milk [DM] from the local human milk bank) during NICU stay, no presence of sepsis. Conversely, infants developing intraventricular hemorrhage grade 3 or 4 (13), periventricular leukomalacia (14), retinopathy of prematurity ≥grade 3 plus disease (15), or necrotizing enterocolitis (NEC) Bell's stage ≥2 (16) during hospitalization were ruled out from the study, in view of the known negative effects of these conditions on growth and development. Globally, 52 ELBW infants were considered eligible for the study.

This study was conducted in conformity with the principles and regulations of the Helsinki Declaration. A written, informed consent to participate was obtained from the parents/guardians of each infant. The protocol was approved by the local Ethics Committees in both the study centers.

### Strategies

The two NICUs had similar protocols for resuscitation, stabilization, ventilation and pharmacological management of ELBW preterm infants, whereas their nutritional approaches were significantly different.

The nutritional protocol of Sant'Orsola Malpighi Hospital's NICU, named Standard Nutrition Protocol (SNP), provided an average protein regimen, administered by combined enteral and parenteral nutrition (PN) according to the European Society of Pediatric Gastroenterology and Nutrition (ESPGHAN) Guidelines (17, 18). This protocol entailed four different phases of enteral nutrition. In phase 1 (minimal enteral feeding [MEF]), minimal milk feeds (10–15 ml/kg/day) were administered to stimulate the anatomical and functional maturation of the gut and to reduce NEC risk (19). During this period, recommended nutrient intakes were guaranteed by PN, started within the first 24 h of life. PN was prescribed according to the ESPGHAN recommendations (17): starting composition consisted of 6 mg/kg/day of glucose and 2–2.5 g/kg/day of aminoacids, which were incremented to 3.5 g/kg/day by day 6. Lipids were introduced from day 3 and gradually increased to 0.5 g/kg/day over the first week of life until the achievement of 3 g/kg/day. Sodium and other electrolytes were added from day 3 onwards and adjusted in relation to serum values and diuresis.

Once feeding tolerance to MEF was obtained, feeds were increased by 15–20 ml/kg/day divided in 8 meals (phase 2) until the achievement of full feeding, defined as enteral volumes of 160 ml/kg/day (phase 3). Human milk fortification was started at volumes of 100 ml/kg/day using Aptamil BMF 4.4% (1,6-1,98 gr proteins/100 ml of milk) at meal administration. If clinical deterioration or symptoms of feeding intolerance (i.e., abdominal distention, absent bowel sounds, persistently bilious or bloody gastric residuals and/or bloody stools) (20), suspected sepsis, NEC or other surgical problems occurred at any phase, enteral feeds were withheld. According to inclusion criteria, 26 ELBW infants were recruited in SNP group.

The nutritional protocol of Bufalini Hospital's NICU was named Aggressive Nutrition Protocol (ANP). According to this protocol, preterm neonates were fed with fresh OMM or DM since their first day of life. Feeds were started at volumes of 10–15 ml/kg/day, divided in 10 meals; when an adequate feeding tolerance was established, feeds were increased by 20–25 ml/kg/die. Protein fortification of HM was started from day 3 (when volume were about 40 ml/kg) onwards at protein intakes of 0.5 g/100 ml of milk with Pro-expert PS (Aptamil) or Protifar (Nutricia), and was incremented to 1% over the next 24 h. When infants tolerated feed volumes of 100 ml/kg/day, BMF (Aptamil) at a concentration of 3 g/100 ml was added to Pro-expert PS, and increased to 5 g/100 ml over the next days. The amount of protein fortification was adjusted according to the newborn's blood urea and acid-base status, monitored twice a week (21): if blood urea was less than 40 mg/dl, protein fortification (Pro-expert PS) was yielded by 1.5%, whereas BMF kept fixed at 5%; no changes were made for levels between 40 and 50 mg/dl and normal acid-base status (pH >7.30 and BE <-4). The maximum level of fortification was obtained with BMF at 5% plus Protifar or Pro-expert PS at 1.5%, depending on the infants' feeding tolerance, blood urea values and acid-base status. According to inclusion criteria, 26 ELBW infants were recruited in ANP group.

### Outcome evaluation

During hospitalization, growth parameters (weight, length and HC), acid-base status, renal function, diuresis, enteral intakes, feeding tolerance, clinical history and the occurrence of clinical complications were regularly assessed and recorded in a clinical report form.

After discharge, as per national recommendations (22), all the enrolled infants were included in the clinical, neurological and neurodevelopmental follow-up of prematurity, which entails a term MRI scan at 40 weeks post-conceptional age and regular evaluations of the infant's growth and neurodevelopmental status up to 24 months CA. At each evaluation, weight, length and HC were measured to assess the infant's growth.

The psychomotor outcome of the enrolled infants was evaluated by two psychologists, with long-standing experience in developmental assessment, blind to the infant's nutrition group, using the Revised Griffiths Mental Development Scales (GMDS-R) 0–2 years (23), which are widely adopted for the evaluation of mental and psychomotor development in the context of the neurodevelopmental follow-up of preterm infants (24–28). These scales investigate five main areas (Locomotor—LOC, Personal-Social - PS, Hearing and Language - HL, Eye-Hand Coordination - EH, Performance—PERF), providing a general quotient (GQ) of the infant's abilities adjusted for corrected age and 5 sub-quotients (SQ) for each functional area.

Data from the 12 and 24-month assessments were included in the present study.

### Statistical analysis

Statistical analysis was performed using Statistical Package for Social Science software for Microsoft Windows (SPSS) version 21.0. Data distribution was checked using Kolmogorov–Smirnov test; all data showed a normal distribution. Clinical characteristics of the study groups were compared by Pearson's chi-squared test and MANOVA.

Possible differences between the study groups in terms of length of NICU stay, growth parameters and in-hospital outcomes were evaluated by MANOVA test. Furthermore, the effects of study group (SNP and ANP), child's age (12 and 24 months CA), and their interaction on anthropometric data and psychomotor scores at post-discharge assessments were tested through Repeated Measure MANOVA. Maternal education and the infant gestational age were included as variables in order to control their possible influence, as emerged in previous literature (9, 24).

Finally, the association among intra-hospital variables and anthropometric data and psychomotor scores at post-discharge assessments was investigated with Pearson correlation coefficients. Fisher test (F) and eta squared (η^2^_p_) values were reported.

Significance level was set at *p* ≤ 0.05.

## Results

A total of 52 neonates were included in the present study, 26 in the SNP group and 26 in the ANP group. The infants' characteristics are detailed in Table 1; while the two study groups were similar in terms of GA and anthropometric characteristics at birth, a significant difference in the distribution of gender, twinhood and mechanical ventilation [MV] was observed. Subsequent analyses showed that infant gender and twinhood did not significantly influence anthropometric data and GMDS scores. For this reason, these variables were not included in further analyses. On the contrary, MV showed a significant association with the outcome scores and was, therefore, controlled in subsequent analyses.

**Table 1:** Biological, socio-demographic and medical characteristics of the two groups.

	**SNP**	**ANP**	***F/X^2^***	***P***
	**(*n* = 26)**	**(*n* = 26)**		
Males, *n* (%)	9 (34.6)	16 (61.5)	3.78	0.05
Birth-weight (g), mean (SD), range	773 (165), 445–1000	826 (136), 692–986	0.11	0.74
Head circumference (cm), mean (SD), range	25.3 (2.55), 2 2–31	24.9 (1.59), 22.8–29	0.59	0.44
Gestational age (weeks), mean (SD), range	27.5 (1.68), 23–31	28.0 (1.75), 2 4–31	0.30	0.59
Cesarean section, *n* (%)	23 (88.5)	21 (80.8)	0.60	0.44
Twinhood, *n* (%)	8 (30.8)	2 (7.7)	4.46	0.03
MV, *n* (%)	13 (50.0)	3 (11.5)	9.03	0.003
SGA, *n* (%)	11 (42.3)	12 (46.2)	0.08	0.78
BPD, *n* (%)	9 (34.6)	5 (19.2)	1.56	0.21
PDA, *n* (%)	11 (42.3)	7 (26.9)	1.36	0.24
Maternal education University, *n* (%)	11 (42.3)	10 (39.1)	2.83	0.24
High school, n (%)	11 (42.3)	7 (26.1)		
Primary and secondary school, *n* (%)	4 (15.4)	9 (34.8)		
Maternal foreign nationality, *n* (%)	4 (15.4)	8 (30.8)	1.73	0.18
Maternal age, mean (SD), range	32.54 (4.37), 24–42	34.55 (4.25), 23–41	2.35	0.13

*SNP, Standard Nutrition Protocol; ANP, Aggressive Nutrition Protocol; MV, mechanical ventilation, SGA, small for gestational age, BPD, bronchopulmonary dysplasia*.

Globally, 60% of the enrolled infants received OMM and 40% pasteurized DM from the local hospital bank.

Eventually, groups were homogeneous for the following maternal characteristics: education, nationality and age (Table 1).

Parenteral and enteral protein intakes for the two groups over the first 4 weeks of life are detailed in Table 2. For each week the daily protein intake was significantly higher in the ANP group compared to SNP group. The high-protein nutritional regimen was well tolerated by the ANP group; no difference in the rate of metabolic acidosis and in serum creatinine levels during NICU stay was seen compared to the SNP group.

**Table 2:** Mean values (standard deviation) of parenteral, enteral and total protein intakes in the two groups during the first 4 weeks of life.

		**SNP**	**ANP**	***F***	***P***
		**(*n* = 26)**	**(*n* = 26)**		
Week 1	Enteral	0.05 (0.05)	0.70 (0.65)	9.73	0.003
	Parenteral	1.39 (0.34)	1.20 (0.48)		< 0.0005
	Total	1.46 (0.36)	1.99 (0.50)	12.77	0.001
Week 2	Enteral	0.39 (0.37)	3.08 (1.27)	48.31	< 0.0005
	Parenteral	1.22 (0.35)	0.10 (0.18)		< 0.0005
	Total	1.69 (0.29)	3.65 (0.99)	41.47	< 0.0005
Week 3	Enteral	1.03 (0.77)	4.22 (0.87)	51.46	< 0.0005
	Parenteral	0.63 (0.52)	0.00 (0.00)		< 0.0005
	Total	1.72 (0.52)	4.22 (0.87)	73.68	< 0.0005
Week 4	Enteral	1.72 (0.99)	4.36 (0.84)	32.66	< 0.0005
	Parenteral	0.64 (0.63)	0.00 (0.00)		< 0.0005
	Total	1.96 (0.71)	4.36 (0.84)	55.36	< 0.0005

*Values describe g/kg/day. SNP, Standard Nutrition Protocol; ANP, Aggressive Nutrition Protocol*.

The rate of infants fed exclusively with OMM at discharge was 62.5% in the ANP group and 65.6% in the SNP group.

### In-hospital outcomes

In-hospital outcomes are detailed in Table 3. Infants in the ANP group showed significantly higher growth rates for weight and HC [*F*_(1, 47)_ = 5.95; *P* = 0.021; *F*_(1, 47)_ = 7.60; *P* = 0.010, respectively], but not for length, when compared to SNP. PN duration and the length of NICU stay were significantly shorter in the ANP group compared to the SNP [*F*_(1, 47)_ = 15.87; *P* < 0.0005; *F*_(1, 47)_ = 21.85; *P* < 0.0005, respectively] (Table 3).

**Table 3:** In-Hospital Outcomes.

	**SNP**	**ANP**	**F/X2**	***P***
	**(*n* = 26)**	**(*n* = 26)**		
Weight gain (gr/kg/day), mean (SD), range	15.20 (2.8), 9.7–23.9	18.87 (3.0), 13.2–25.0	5.95	0.021
Length growth (cm/week), mean (SD), range	0.95 (0.5), 0.1–2.3	0.88 (0.2), 0.3–1.4	0.62	NS
Head circumference growth (cm/week), mean (SD), range	0.52 (0.2), 0.2–0.9	0.70 (0.2), 0.1–1.1	7.60	0.010
Milk volume, ml/kg, mean (SD), range	150.52 (17.6), 115.6–200.9	160.7 (7.3), 149.3–198.8	0.12	NS
Days of PN, mean (SD), range	38.75 (29.6), 9–106	7.36 (2.7), 2–14	15.86	< 0.0005
Days to achieve full feeding, mean(SD), range	28.50 (17.2), 9-78	7.50 (2.0), 4-12	28.40	< 0.0005
Weight at full feeding, mean(SD), range	895.42 (294.8), 535–1620	748.8 (112.70),526–940	2.68	NS
Days of hospitalization, mean (SD), range	78.08 (21.32),46–145	60.0 (13.3),31–81	21.85	< 0.0005
EUGR, n (%)	22 (84.6%)	16 (61.5%)	3.52	0.06

*SNP, Standard Nutrition Protocol; ANP, Aggressive Nutrition Protocol; PN, Parenteral Nutrition; EUGR, Extra-Uterine Growth Restriction*.

### Post-discharge outcomes

No pathological findings at term MRI were observed in the infants enrolled. At 24 months CA, no child developed cerebral palsy.

Anthropometric measures at 12 and 24 months CA in the two groups are provided in Table 4. Repeated measure MANOVA showed no significant effect of the Study Group on weight and length between two groups, whereas head circumference was significant higher in the ANP than in SNP group [SNP mean = 45.64 ± 0.29; ANP mean = 46.80 ± 0.31; *F*_(1, 40)_ = 7.844; *P* = 0.008]. A significant interaction between Study Group and Child's Age emerged for the children length [*F*_(1, 40)_ = 27.170; *P* < 0.0005]: at 24 months CA SNP children were taller than ANP ones (*P* = 0.04), whereas no significant difference for weight and head circumference was observed.

**Table 4:** Anthropometric data.

	**SNP** (***n*** = **26)**		**ANP** (***n*** = **26)**		**RM-MANOVA**		
	**Mean(SD)**	**Range**	**Mean(SD)**	**Range**	***F***	***P***	***η p^2^***
Weight (g*)*					2.509	0.121	0.059
12 mo. ca	8027.7 (1109)	5915–10660	8546.15 (1175.8)	6800–10900			
24 mo. ca	10375.8 (1363)	8110–13700	10572.31 (1280.7)	8530–13200			
Length (cm)					27.170	< 0.0005	0.405
12 mo. Ca	72.3 (3.14)	68–81	72.9 (2.79)	67–78			
24 mo. ca	84.7 (3.20)	80–92	82.2 (3.15)	76–89			
HC (cm)					2.985	0.092	0.069
12 mo. ca	44.9 (1.46)	41–47	45.8 (1.57)	43–48			
24 mo.ca	47.15 (1.25)	44.3–49.4	47.8 (1.24)	45.5–50			

*The table summarizes mean, SD, range, F, p and eta-squared (?2p) values for MANOVAs on Anthropometric data of SNP (Standard Nutrition Protocol) and ANP (Aggressive Nutrition Protocol) infants at 12 and 24 months (corrected age)*.

**Figure 1:**
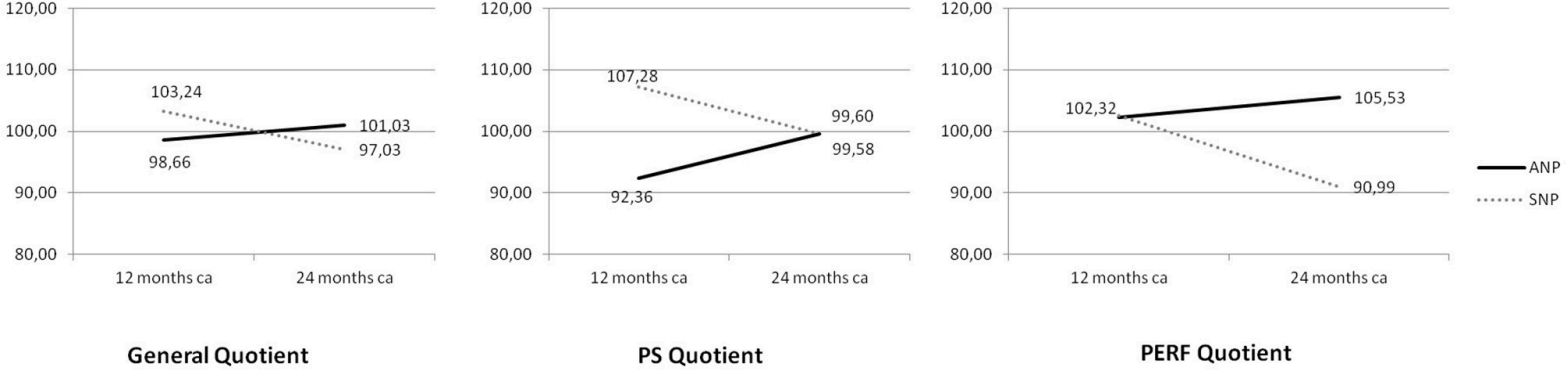
Mean scores on General Quotient (GQ), Personal and Social skills (PS), and Performance (PERF) according to the interaction between Study Group and Child's Age. Continue line denotes ANP (Aggressive Nutrition Protocol) group; dotted line SNP (Standard Nutrition Protocol) group.

Psychomotor data at 12 and 24 months CA in the two groups are provided in Table 5. Mean values of GQ and SQ scores fell within normal ranges^22^ in both groups at 12 and 24 months except for PERF mean values in the SNP group at 24 months, which fell below the lower normal threshold. Despite no significant differences between SNP and ANP emerged, the interaction between Study Group and Child's Age significantly influenced the GQ [*F*_(1, 40)_ = 9.062; *P* = 0.005] and the following SQ: PS [*F*_(1, 40)_ = 10.743, *P* = 0.002], and PERF [*F*_(1, 40)_ = 6.653, *P* = 0.014] (Figure 1). Bonferroni *post hoc* analyses showed that QG, PS and PERF mean scores significantly decreased from 12 to 24 months CA [*P* = 0.003; *P* = 0.018; *P* = 0.006, respectively] but only in SNP group; moreover, PS quotients of ANP children (but non SNP ones) significantly increased from 12 to 24 months CA [*P* = 0.047] (Figure 1). Eventually, when compared to SNP, ANP children showed higher PERF scores at 24 months CA [*P* = 0.013] (Figure 1).

**Table 5:** Infant's psychomotor mean scores at post-discharge assessments.

	**SNP** (***n*** = **26)**		**ANP** (***n*** = **26)**		**RM-MANOVA**		
	**Mean(SD)**	**Range**	**Mean(SD)**	**Range**	***F***	***P***	***η p^2^***
GQ score, mean (SD), range					9.062	0.005	0.197
12 mo. ca	103.24 (10.66)	72–119	97.03 (11.79)	66–117			
24 mo. ca	97.03 (11.77)	68–113	101.03 (110.76)	81–120			
LOC score, mean (SD), range					3.130	0.085	0.078
12 mo. Ca	95.28 (16.70)	57–117	97.79 (16.38)	57–121			
24 mo. ca	98.94 (19.30)	60–135	113.93 (20.59)	60–135			
PS score, mean (SD), range					10.743	0.002	0.225
12 mo. ca	107.28 (13.60)	64–122	92.36 (13.20)	56-114			
24 mo.ca	99.60 (13.45)	59–119	99.58 (12.87)	66-119			
HL score, mean (SD), range					0.279	0.601	0.007
12 mo. ca	107.28 (17.08)	85–150	102.55 (13.26)	74–129			
24 mo.ca	99.64 (18.43)	50–115	92.53 (14.43)	50–113			
EH score, mean (SD), range					2.825	0.101	0.071
12 mo. ca	102.08 (14.55)	79–128	97.22 (15.93)	68–122			
24 mo.ca	98.89 (14.28)	71–123	103.62 (14.27)	76–123			
PERF score, mean (SD), range					6.653	0.014	0.152
12 mo. ca	102.32 (12.09)	76–122	102.32 (13.12)	63–123			
24 mo.ca	90.99 (18.74)	50–117	105.53 (12.37)	77–121			

*The table summarizes mean, SD, range, F, p and eta-squared (${?}_{\text{p}}^{2}$) values for MANOVAs on GQ, General Quotient; LOC, Locomotor; PS, Personal & Social skills; HL, Hearing & Language; EH, Eye & Hand Coordination; and Performance (PERF) standardized scores (GMDS-R) of SNP (Standard Nutrition Protocol) and ANP (Aggressive Nutrition Protocol) infants at 12 and 24 months (corrected age)*.

No significant effect of Child's Age emerged on anthropometric nor psychomotor outcomes.

Protein intakes in the first 2 weeks of life positively correlated with HC at 24 months CA (*P* = 0.031). No significant correlation between protein intakes in the first 2 weeks and GMDS quotients were observed.

## Discussion

According to our results, higher protein intakes over the first 4 weeks of life in ELBW infants are associated with improved growth of HC and psychomotor outcomes at 24 months CA, thus highlighting the importance of in-hospital nutrition. Moreover, the present data confirm that adjustable fortification of HM combining different commercially available concentrated HM fortifiers effectively allows the achievement of protein intakes and protein/energy ratio currently recommended for the ELBW population during the first weeks of life.

While more is known about recommended protein intakes for very-low-birth-weight infants, little data are currently available for ELBW babies. Basing on empirical calculations, an enteral protein intake of 4.0–4.5 g/kg/day is currently recommended for infants up to 1000 g of weight to prevent protein deficit accumulation and to aim at growth patterns similar to intrauterine ones (18). Particular attention should be paid also at protein/energy ratio that, for infants <1000 g, ranges from 3.2 to 4.1 g/100 kcal. In growth-restricted infants, energy intakes can be increased; however, if not accompanied by adequate protein intakes, growth is achieved at the expenses of body composition, ensuing in a high percentage of body fat (19) that could contribute to worsen the increased intraabdominal adiposity observed in ELBW neonates and to heighten their risk of metabolic complications in later life (29).

Assuming HM protein contents between 0.8 and 1.2 g/100 ml (10, 11), the estimated protein intakes at maximum fortification levels and at enteral intakes of 160 ml/kg/day were 3.5 g/kg/ day in the SNP group and 4.8 g/kg/day in the ANP group, whereas the protein/energy ratio (protein g/100 Kcal) ranged between 1.9–2.3 and 3.0–3.3, respectively. Hence, according to the above recommendations, the estimated protein requirements were met in the ANP but not in the SNP group; consistently, the latter showed higher EUGR rates.

In 2013, Cormack et al. (30) investigated the effects of protein intake equal or greater to 4 g/kg/day provided during the first week of life in a predominantly HM fed cohort of ELBW babies, reporting a significant association between protein intake and in-hospital growth: the higher the intake, the smaller the z-score change between birth and discharge.

In addition to the beneficial effects on in-hospital outcomes and head circumference growth at 12 and 24 months CA, the present study has demonstrated increased length after discharge. However, an unattended result regards the outcome of length, where ANP infants obtained worse scores than those of SNP group. According to previous researches (27, 28), length was measured to the nearest cm using a length board: it could be possible this kind of measure is not adequately sensitive and has a increased risk of measurement bias. Further studies are needed to better explain this result.

Our results are in line with Stephen et al. (12), who had previously described a positive correlation between increased first-week protein and energy intakes and higher Mental Development Index scores at 18 months CA in an ELBW population. Despite ANP and SNP did not differ in the GMDS quotients mean scores, the trajectory of these scores is significantly different in the scales, with a lower decrement and a better psychomotor development at 24 months CA in ELBW infants receiving early and high protein intakes. Specifically, ANP children not only show better personal-social skills outcome at 24 months, but their GQ, PS and PERF mean quotients do not decrease as emerged in SNP group. Despite preliminary, this result is promising: future studies could deepen if this intervention reduce the negative effect of severely preterm birth on long term development. The Hearing and Language quotients is the only where effects did not emerged, confirming as this area is particularly weak for very preterm infants (24, 26, 31).

Taken together, these results suggest a long-lasting beneficial influence of this protein regimen on cerebral maturation.

Recently, a Cochrane review investigating the effect of high protein intake in formula-fed low-birth-weight infants has reported elevated blood urea nitrogen levels and an increased incidence of metabolic acidosis in association with protein intakes ranging between 3 and 4 g/kg/day (32). However, little is known about the incidence of these adverse effects in HM-fed ELBW infants. In the present study, the ANP group did not show increased blood urea or higher rates of metabolic acidosis, suggesting that this high-protein regimen was adequately tolerated by the study ELBW population.

Although a trend toward an earlier introduction of enteral feeds has occurred over the last few years, ELBW infants often do not begin enteral nutrition for several days and do not reach full feeding for weeks. In the ANP population, enteral feeds were introduced since the first day of life and faster rates of feeding advancement were adopted, resulting in earlier full feeding achievement, shortened PN duration -related complications and a significantly briefer length of hospitalization. The combination of these factors may have contributed to the improved psychomotor outcomes of ANP infants at 24 months CA; however, the design of the present study did not allow to define the role of each factor in determining the observed outcome.

A number of limits could be acknowledged for the present study. Firstly, the results need to be confirmed on wider samples. Secondly, a significant difference in the distribution of a clinical complication (mechanical ventilation) emerged. Despite this variables was controlled in analyses, results need replication. Thirdly, the small sample size did not allow to focus on SGA infants, which are known as a high risk population (32). Further studies are therefore recommended which should consider also these factors.

In conclusion, a high-protein regimen, associated with an early introduction and advancement of exclusive HM feeds, can lead to improved in-hospital growth, lower rates of EUGR, shorter length of NICU stay and better psychomotor and growth outcomes at 24 months CA in ELBW preterm infants. Further larger studies are needed to confirm these preliminary data, to assess the contribution of different nutrient components and other clinical or environmental variables on post-discharge growth and neurodevelopment and to investigate possible long-term effects of high-protein regimens in this high-risk population.

## Author contributions

EM and AB prepared the study design, organized the sample recruitment, collected data, and contributed to the writing of the manuscript's introduction, discussion, and references sections. LM, SM, AA, and SS contributed to the recruitment of the sample and to data collection. LC, GF, AS, FA, and MS contributed to prepare the study design and supervised data collection and the research team. EN performed statistical analysis, prepared the tables, and contributed to write all sections of the manuscript. All authors reviewed and approved manuscript for publication.

### Conflict of interest statement

The authors declare that the research was conducted in the absence of any commercial or financial relationships that could be constructed as a potential conflict of interest. The handling editor declared a shared affiliation, though no other collaboration, with one of the authors AB.
